# Association study of newly identified age-related macular degeneration susceptible loci *SOD2, MBP,* and *C8orf42* in Han Chinese population

**DOI:** 10.1186/1746-1596-9-73

**Published:** 2014-03-25

**Authors:** Mengyuan Kan, Fatao Liu, Xiaoling Weng, Junyi Ye, Ting Wang, Mingqing Xu, Lin He, Yun Liu

**Affiliations:** 1 Institute for Nutritional Sciences, Shanghai Institutes for Biological Sciences, Chinese Academy of Sciences, Graduate School of the Chinese Academy of Sciences, Shanghai 200031, P. R. China; 2 Institutes of Biomedical Sciences, Fudan University, Ming Dao Building, 138 Yi Xue Yuan Road, Shanghai 200032, P. R. China; 3 Bio-X Institutes, Key Laboratory for the Genetics of Developmental and Neuropsychiatric Disorders (Ministry of Education), Shanghai Jiao Tong University, Shanghai 200030, P.R. China

**Keywords:** Age-related macular degeneration, Han Chinese population, Association study

## Abstract

A recent genome-wide association study has reported three newly identified susceptible loci (rs2842992 near the gene *SOD2*, rs1789110 near the gene *MBP* and rs722782 near the gene *C8orf42*) to be associated with the geographic atrophy subtype of age-related macular degeneration in European-descent population. We investigated the correlation between these variants and advanced age-related macular degeneration for the first time in a Han Chinese cohort; however, no evidence supports these previously identified loci contribute to advanced age-related macular degeneration susceptibility in Chinese population.

## Letters to the editor

Age-related macular degeneration (AMD) is a late-onset neurodegenerative disease, which is the major cause of visual impairment and blindness in adults aged 55 years or older [1]. AMD is characterized with formation of choroidal drusen, yellow deposits between the retinal pigment epithelium (RPE) and underlying choroid in the early stage. The progression of AMD in an advanced stage (advanced AMD) can be classified as atrophy of the RPE (geographic atrophy (GA), or dry AMD), or as the growth of abnormal choroidal vessels under the retina (choroidal neovascularization (CNV), or wet AMD) [2,3]. Previous studies showed that the estimated prevalence of advanced AMD was 1.0% in Chinese compared to 0.6% in Europeans [4] and CNV form was more common in Chinese individuals [4,5]. Genetic studies often combined GA and CNV cases as overall advanced AMD to find the heritable loci and some subgroup analysis also suggested that these two subtypes of advanced AMD might share common genetic risk factors [6-10]. Recently, a genome-wide association study (GWAS) has found several single nucleotide polymorphisms (SNPs) specifically associated with GA subtype or CNV subtype in European-descent population [11]. We aimed to investigate SNPs which were correlated with GA (rs2842992 near *SOD2* (P = 3.4 × 10^-7^); rs1789110 near *MBP* (P = 4.2 × 10^-7^) and rs722782 near *C8orf42* (P = 1.9 × 10^-6^)) in a Han Chinese cohort, given a sample with higher proportion of GA subtype (37.7% for GA versus 26.5% for CNV). Due to the small sample size for each subtype (77 GA cases, 54 CNV cases and 73 advanced AMD cases lacking of further subtype information), we combined the cases as a whole and analyzed the three SNPs to determine any possible effect on advanced AMD risk in Chinese population.

204 AMD cases were recruited from the Putuo People’s Hospital in Zhoushan, China and 384 unrelated healthy controls were enrolled from the same region. AMD clinical diagnosis was defined strictly in accordance with the International Classification of Age-Related Maculopathy and Macular Degeneration [2]. A standard informed consent was established following the guidelines of the Helsinki Declaration and approved by the ethics committee of the Shanghai Institute for Biological Sciences. All the participants obtained and signed the consent. A demographic summary of the phenotypic information was shown in Table 1. High-molecular-weight genomic DNA was prepared from venous blood using standard phenol-chloroform extraction. The variants of interest were genotyped by TaqMan SNP genotyping assay (Life Technologies, Carlsbad, California, USA). Genotype data were obtained in more than 93% of the DNA samples and 38 replicated quality controls were genotyped with 100% concordance. We calculated Hardy-Weinberg equilibrium for each variant based on an exact test [12] and no departures from the equilibrium were observed in controls (P > 0.05). We evaluated the association between alleles and AMD in terms of odds ratios (ORs), 95% confidence intervals (CIs), and corresponding P values based on Fisher’s exact test. We further assessed the association between genotypes and AMD using logistic regression assuming an additive model. As we found significant difference of age distributions between cases and controls (P < 0.001), we also analyzed the genotypic associations with adjustment for age. All of the statistical tests were carried out using the R software package (http://www.r-project.org/), and a P-value < 0.05 was defined as statistical significance. Statistic powers was *post hoc* calculated using the G*Power program, based on goodness-of-fit test [13].

**Table 1:** Characteristics of the participants

**Characteristics**	**Case**	**Control**
N	204	384
GA subtype (%)	37.7	/
CNV subtype (%)	26.5	/
AMD diagnosis (%)^a^	35.3	/
Female (%)	44.2	55.9
Age (years)	74.9 ± 7.0 (53–89)	67.6 ± 6.6 (52–86)

*Abbreviations:GA* geographic atrophy, *CNV* choroidal neovascularization.

Data are presented as the mean ± standard deviation with range (minimum to maximum).

^a^These cases were diagnosed with AMD, but we could not acquire specific subtype information.

The allelic and genotypic association results for the selected variants (rs2842992, rs1789110 and rs722782) were shown in Table 2. We did not find evidence of association between these variants and advanced AMD either in allele level (P = 0.277 for rs2842992; P = 0.850 for rs1789110 and P = 0.281 for rs722782) or genotype level (P = 0.266 for rs2842992; P = 0.798 for rs1789110 and P = 0.279 for rs722782) in our samples. Even after correction for age, the associations with advanced AMD still remained insignificant (P = 0.661 for rs2842992; P = 0.537 for rs1789110 and P = 0.080 for rs722782). Moreover, we observed little heterogeneity between GA cases and non-GA cases as the effective allele frequencies are very close in these two groups (Table 2).

**Table 2:** Allelic and genotypic associations between three selected SNPs and AMD

**SNP information**				**Allelic associations**						**Genotypic associations**				
**dbSNP ID**	**Gene**	**chr:pos**	**Location**	**Alleles**^**a**^	**Effective allele frequency**				**P value**^**b**^	**Genotypic distributions**			**P value**^**c**^	**P value**^**d**^
									**OR (95% CI)**				**OR (95% CI)**	**OR (95% CI)**
rs2842992	*SOD2*	6:160071159	Intergenic	**A**/G	Case	0.523	GA case	0.547	0.277	AA/AG/GG	Case	47/107/38	0.266	0.661
					Control	0.558	Non-GA case	0.508	0.87 (0.67-1.13)		Control	109/165/69	0.87 (0.67-1.12)	0.93 (0.68-1.28)
rs1789110	*MBP*	18:74859044	Intergenic	**A**/C	Case	0.542	GA case	0.533	0.850	AA/AC/CC	Case	55/107/38	0.798	0.537
					Control	0.535	Non-GA case	0.548	1.03 (0.80-1.33)		Control	100/171/76	1.03 (0.80-1.33)	1.10 (0.81-1.51)
rs722782	*C80rf42*	8:516479	Intergenic	**A**/C	Case	0.473	GA case	0.471	0.281	AA/AC/CC	Case	43/92/53	0.279	0.080
					Control	0.508	Non-GA case	0.475	0.87 (0.67-1.12)		Control	97/177/91	0.87 (0.68-1.12)	0.76 (0.56-1.03)

*Abbreviations*: *chr:pos* chromosome:position, *OR* odds ratio, *CI* confidence interval.

^a^Effective alleles are indicated in bold type, and odds ratios were calculated based on this allele.

^b^ORs, 95% CIs and P values were calculated based on Fisher’s exact test.

^c^ORs, 95% CIs and P values were calculated using logistic regression, assuming the additive model.

^d^ORs, 95% CIs and P values were calculated using logistic regression, assuming the additive model with adjustment for age.

One explanation for the failure to replicate the associations in our study could be the phenotypic difference between GA and CNV of advanced AMD: GA is characterized by the loss of normal RPE cells in a discrete area of the macula (≥ 175 μm in diameter) with a sharp border and visible choroidal vessels in the absence of CNV, which has some clinic aspects in common with chloroquine retinopathy [14]; while CNV is featured by haemorrhagic detachment of RPE and presence of subretinal fibrous, with the development of neovascularisation towards the macula resulting in leaking fluid or blood [2,3]. As these loci were identified to be associated with GA alone by Sobrin et al. [11], while we used overall advanced AMD which included both GA and CNV cases, it is possible that the associations were masked by the phenotypic heterogeneity of mixture of GA and CNV in our samples. Another explanation for the negative findings could be the low statistical powers in our study (21.7% for rs2842992, 5.5% for rs1789110 and 21.4% for rs722782), which indicated inadequate powers to detect potential associations. However, if the disease risk could reach a modest effect (OR, 0.70) as reported by Sobrin et al. [11], we found the sample size had > 90% power for these variants to detect the association. Furthermore, in previous GWAS study [11], these loci were reported to be highly associated with GA (P values ranging from 10^-6^ to 10^-7^), but none of them reached the generally used genome-wide threshold (P = 5.0 × 10^-8^), thus these loci were only considered as suggestive association signals by the authors. Meanwhile, the discrepancies of genetic structures and disease prevalence among populations may also account for the inconsistency.

Among these three AMD genetic loci (*SOD2*, *MBP* and *C8orf42*) [11], *SOD2* was previously studied with a common missense variant rs4880 which showed associations with AMD in Japanese population [15], while this finding was inconsistent with follow-up studies [16-18]. *SOD2* gene encodes a mitochondrial enzyme, which is involved in the detoxification of superoxide free radicals. Oxidative damage to the retina, RPE or choroid complex is implicated for the development of AMD [19,20]. Functional studies demonstrated SOD2 (superoxide dismutase 2) protects against reactive oxygen species-induced oxidative damage in mouse RPE [20,21], indicating a role in AMD pathogenesis. Aside from *SOD2*, currently there is no other genetic evidence for *MBP* and *C8orf42* loci contributing to AMD, and whether these genes have functional potentiality to AMD pathogenesis remains to be elucidated.

In summary, the SNPs on genetic loci *SOD2*, *MBP* and *C8orf42*, which predisposed to GA risk in European population, were not found significantly associated with advanced AMD in a Chinese cohort. Additional studies with larger sample size and more makers are important to clarify whether these loci confer a risk of overall advanced AMD or AMD subtypes in Chinese population.

## Abbreviations

AMD: Age-related macular degeneration; RPE: Retinal pigment epithelium; GA: Geographic atrophy; CNV: Choroidal neovascularization; SNP: Single nucleotide polymorphism; GWAS: Genome-wide association study; OR: Odds ratios; CI: Confidence interval; SOD2: Superoxide dismutase 2.

## Competing interests

The authors declare no competing interests.

## Authors’ contributions

YL and LH conceived the study; MK conducted the experiments, analyzed the data, and drafted the manuscript; FL analyzed the data; XW and TW conducted the experiments; MX revised the draft. All the authors have read and approved the final manuscript.
